# Patients with Adult-Onset Still’s Disease in Germany: A Retrospective Analysis of Clinical Characteristics and Treatment Practices Ahead of the Release of the German Recommendations

**DOI:** 10.3390/jcm14030981

**Published:** 2025-02-04

**Authors:** Verena Schoenau, Sarah Wendel, Koray Tascilar, Joerg Henes, Eugen Feist, Niklas Thomas Baerlecken, Florian Popp, Matthias Schmidt-Haendle, Bernhard Hellmich, Ina Kötter, Ioana Andreica, Jürgen Rech

**Affiliations:** 1 Department of Internal Medicine 3—Rheumatology and Immunology, Friedrich-Alexander University (FAU) Erlangen-Nürnberg and Universitätsklinikum Erlangen, 91054 Erlangen, Germany; 2 Deutsches Zentrum Immuntherapie, Friedrich-Alexander University (FAU) Erlangen-Nürnberg and Universitätsklinikum Erlangen, 91054 Erlangen, Germany; 3 Department of Rheumatology and Clinical Immunology, Medical Center—University of Freiburg, Faculty of Medicine, University of Freiburg, 79106 Freiburg im Breisgau, Germany; 4 Centre for Interdisciplinary Clinical Immunology, Rheumatology and Auto-inflammatory Diseases, Department of Internal Medicine II (Oncology, Haematology, Immunology and Rheumatology), Universitatsklinikum Tübingen, 72074 Tübingen, Germany; 5 Helios Clinic Vogelsang-Gommern, Department for Rheumatology and Clinical Immunology & Experimental Rheumatology, Otto-von-Guericke-University, 39106 Magdeburg, Germany; 6 Private Practice Rheumatology, 50996 Cologne, Germany; 7 MVZ for Rheumatology Dr. M. Welcker, 82152 Planegg, Germany; 8 Rheumapraxis Bayreuth, 95444 Bayreuth, Germany; 9 Medius Kliniken, Department of Internal Medicine, Rheumatology, Pulmonology, Nephrology and Diabetology, University of Tübingen, 73230 Kirchheim-Teck, Germany; 10 University Hospital Hamburg-Eppendorf, 20246 Hamburg, Germany; 11 Clinic for Rheumatology and Immunology, 24576 Bad Bramstedt, Germany; 12 Rheumatology, Ruhr-Universität Bochum, 44801 Bochum, Germany; 13 Rheumazentrum Ruhrgebiet, 44649 Herne, Germany; 14 Center for Rare Diseases Erlangen (ZSEER), Friedrich-Alexander University (FAU) Erlangen-Nürnberg and Universitätsklinikum Erlangen, 91054 Erlangen, Germany

**Keywords:** adult-onset Still’s disease, Still’s syndrome, retrospective study, patient characteristics, disease activity, treatment, interleukin-1 inhibitors, autoinflammatory disorder

## Abstract

**Background/Objectives:** Adult-onset Still’s disease (AOSD) is an autoinflammatory disorder that can be challenging to diagnose and manage. The aim of this study was to analyze retrospective data to provide insights into the clinical presentation, disease activity, and treatment patterns and outcomes of AOSD during routine clinical care prior to the release of new AOSD guidelines. **Methods:** This retrospective database analysis evaluated adult patients (≥18 years) with a diagnosis of AOSD who engaged in a clinical visit between 1 January 2010 and 31 December 2020. The evaluated outcomes included demographic characteristics, symptoms, disease activity, and treatment. **Results:** Our study included 120 patients (67 [55.8%] of whom were female) diagnosed with AOSD according to the Yamaguchi criteria at ten German rheumatology centers. The median (quartile [Q] 1, Q3) age was 51 (36, 62) years, and the median (Q1, Q3) time from diagnosis was 9 (4, 11) years. Approximately half (66 [55.0%]) had a polycyclic disease course. The most frequent symptoms at initial diagnosis were arthralgia (105 [87.5%]) and fever (86 [71.7%]), and these symptoms continued for a substantial proportion of patients at the current visit (35 [29.2%] and 22 [18.3%], respectively). High neutrophil and ferritin levels were also common. The mean Still Activity Score, a measure of disease activity, improved from 4.66 at initial diagnosis to 1.97 at the most recent visit. The treatments most frequently used at some point in the disease course were glucocorticoids (118 [98.3%]), interleukin (IL)-1 inhibitors (89 [74.2%]), and methotrexate (85 [70.8%]). The most common current treatments were IL-1 inhibitors (55 [45.8%]), followed by methotrexate (29 [24.2%}) and glucocorticoids (28 [23.3%]). **Conclusions:** Our cohort of patients with AOSD seen at German rheumatology clinics showed strong improvements in symptoms and disease activity from initial diagnosis, but a high symptom burden remained for some patients. Future studies may be able to build on our data to document the impact of new guidelines on treatment patterns.

## 1. Introduction

Adult-onset Still’s disease (AOSD) and systemic juvenile idiopathic arthritis (sJIA), the latter of which is now considered to be an earlier form of AOSD [1,2], constitute Still’s syndrome, a rare polygenic autoinflammatory disorder. The incidence of AOSD is reported to be between 0.1 and 0.4 per 100,000 adults [3]. While AOSD typically manifests around the age of 35, cases have been recorded where the onset occurs past the age of 70 [4]. AOSD is associated with a reduced quality of life and substantial economic costs, particularly for patients experiencing significant complications [5,6].

AOSD was first described by Bywaters in 1971 [7], and its primary clinical symptoms include a high fever, a transient salmon-colored rash, oligoarticular arthritis or arthralgia, a sore throat, and hepatomegaly and/or splenomegaly, often accompanied by laboratory abnormalities [8]. Laboratory findings typically reveal systemic inflammation with elevated levels of C-reactive protein (CRP) and a higher erythrocyte sedimentation rate (ESR), significantly increased ferritin levels (3–5 times above normal), and leukocytosis, primarily with neutrophilia. Other laboratory variables, including interleukin (IL)-18, glycosylated ferritin, and proteins such as S100A8/A9 and S100A12, are currently under investigation as potential biomarkers but are not routinely evaluated [9,10,11]. Three predominant disease patterns have been identified: a monocyclic course, which typically resolves within a year of presentation with no relapses; a polycyclic course characterized by unpredictable periods of symptoms separated by months or years; and a chronic, progressive course [8,12]. Some studies also suggest that AOSD can be grouped into two phenotypes, a systemic form and a chronic articular form that evolves into a disease resembling rheumatoid arthritis [8,13].

Despite extensive research, the exact etiology of AOSD remains unknown [8]. It has been proposed that infections or cellular stress lead to an activation of the innate immune system via pathogen-associated molecular patterns or damage-associated molecular patterns. These events, in turn, result in the production of alarmins (e.g., S100 proteins) and the subsequent overproduction of key inflammatory mediators such as IL-1β, IL-6, tumor necrosis factor (TNF), and IL-18 [10,11,14]. If this process is not adequately controlled, chronic inflammation may ensue, leading to a transition from systemic inflammation to a chronic arthritis-driven form of the disease that is sustained by the adaptive immune system [11,15].

Diagnosis of AOSD can be difficult and is often delayed. A recent study reported that the mean time from the development of first symptoms to AOSD diagnosis in German rheumatology clinics was 15.6 months [16], and other centers have reported similar delays [13], although the length varies widely by site. Diagnosis is typically based on a combination of clinical and laboratory findings and requires the exclusion of infections, malignancies, and other inflammatory conditions, including macrophage activation syndrome (MAS), hemophagocytic lymphohistiocytosis, vasculitides, and other periodic fever syndromes [8,10,17,18,19]. Rare but severe complications of AOSD include MAS [20,21], lung disease [22,23], and amyloidosis in cases of chronic inflammation [24].

The development of therapy and treat-to-target (T2T) protocols for AOSD has been challenging due to a need for larger and more rigorous comparative studies of treatment options in AOSD as well as for standardized definitions of a therapeutic response [1]. Although disease activity scores have been applied to AOSD, particularly the Pouchot score [25,26] and Still Activity Score (SAS) [27], there is currently no consensus on which efficacy outcome measure is the best indicator of disease activity [28], and disease activity measures are not uniformly used in clinical studies [29]. The European Alliance of Associations for Rheumatology (EULAR) has recently developed an AOSD disease activity score, the DAVID score [30], which may allow more consistent assessments of AOSD and better characterization of treatment responses.

The recently released joint EULAR and Paediatric Rheumatology European Society (PReS) guidelines recommend treatment with IL-1 inhibitors (anakinra or canakinumab) or IL-6 receptor (IL-6R) inhibitors (tocilizumab) for patients with sJIA/AOSD; for patients with high disease activity, concomitant glucocorticoid administration may be required to achieve disease control and tapered when possible [1]. Similar management recommendations have been made by an expert panel [31]. The German Society of Rheumatology S2 guidelines are generally consistent with these recommendations [32]. However, one key difference is that the EULAR/PReS guidelines prioritize IL-1 and IL-6R inhibitors over conventional synthetic disease-modifying antirheumatic drugs (csDMARDs), such as methotrexate (MTX), for glucocorticoid-sparing and initial therapies regardless of disease activity [1], whereas the German Society of Rheumatology S2 guidelines include MTX or calcineurin inhibitors (cyclosporine) along with IL-1 and IL-6R inhibitors as considerations for glucocorticoid-sparing first-line agents for patients with mild disease activity [32]. The ultimate goal of treatment is drug-free remission with no AOSD-related symptoms and normal levels of CRP and ESR [1].

Attaining a better understanding of possible improvements in symptomatic burden and treatment outcomes associated with the recent AOSD guidelines requires baseline data on patient characteristics, disease activity, and treatment patterns prior to the introduction of the guidelines. This study aimed to retrospectively collect and analyze data from multiple rheumatology centers across Germany to better understand the clinical presentation, disease activity, and treatment outcomes for patients with AOSD in the years preceding the release of the new EULAR and German Society of Rheumatology guidelines.

## 2. Materials and Methods

### 2.1. Study Design and Setting

This study was a retrospective analysis of data obtained from patients with AOSD seen at German rheumatology centers in the following cities: Augsburg, Bad Bramstedt, Erlangen, Freiburg, Gommern, Herne, Kirchheim Teck, Köln, Planegg, and Tübingen. The primary goal of this study was to descriptively evaluate current AOSD characteristics and management practices in Germany. All included patients had a confirmed diagnosis of AOSD according to the validated Yamaguchi criteria [33,34], i.e., the diagnostic criteria recommended by the German Society of Rheumatology S2 guidelines [32], at the time of data collection (between 1 January 2010, and 31 December 2020) or documented disease recurrence. Patients were required to be adults at the time of data collection and willing to provide pseudonymized data. There were no other inclusion or exclusion criteria. Data for current status were collected at a single visit; medical records were used to obtain data on each patient’s status at initial diagnosis and any therapies they had ever received.

Data collection was conducted following approval by the central ethics committee of Erlangen University (Ethics Approval: 365_20 Bc), and the data were anonymized to ensure patient confidentiality. All patients provided informed consent.

### 2.2. Outcomes

The evaluated patient variables were based on data entered by a clinician during routine clinical practice. Patient data included demographic features, clinical manifestations, the presence of comorbidities/complications associated with the disease or its treatment (e.g., MAS, osteoporosis, diabetes, hypertension, and fractures), and disease pattern (monocyclic, polycyclic, or chronic). Disease activity was assessed using the Pouchot score, ranging from 0 to 12 [25,26], and the Still Activity Score (SAS), ranging from 0 to 7 [27], which were calculated based on symptoms, organ involvement, and laboratory values. For both scales, lower scores indicate less disease activity. Medical records were used to extract data on these outcomes at the time of initial diagnosis to allow evaluation of the disease course over time. Patient-reported global disease activity (PtGA) was measured using a visual analog scale (VAS) on a scale of 0 (best) to 10 (worst). Patient satisfaction with therapy was measured on a VAS from 0 (worst) to 10 (best). Current treatment strategies as well as any AOSD therapies ever received were reported. Laboratory values used for diagnosis, specifically ferritin levels ≥ 350 ng/mL and neutrophils accounting for ≥65% of white blood cells, were also evaluated. The database did not include additional laboratory values, such as ESR or CRP.

### 2.3. Statistical Analysis

As this was a descriptive study, sample size calculations were not performed; the sample size was based on all patients who met the inclusion criteria. Descriptive data, including numbers with percentages, means with standard deviations (SDs), and medians with the quartile (Q) 1–Q3 range, were calculated for specified observed outcomes. Comparisons between baseline and follow-up characteristics were made using the McNemar test for categorical characteristics and the paired t-test for numerical variables. Comparisons between males and females were made using the Wilcoxon rank-sum test and the Fisher or Chi-squared test based on the variable type. Missing data were not imputed. Statistical analyses were conducted using the open-source software product R (V 4.0, R Foundation for Statistical Computing, Vienna, Austria). *p* values less than 0.05 were considered significant.

## 3. Results

Ten centers and 120 AOSD patients participated in this study. Over half (55.8%) of the patients were female, and 44.2% were male (Table 1). The mean (SD) age at diagnosis was 41.4 (16.8) years, and the mean (SD) current age was 50.7 (16.2) years. The mean (SD) time since diagnosis was 9.4 (6.9) years. Most patients (71.7%) were being seen at the rheumatology center during a follow-up visit following an earlier diagnosis, while 28.3% were being seen due to disease recurrence. The most common disease course was polycyclic (55.0%), followed by chronic (24.1%) and monocyclic (20.8%). The most common current comorbidities or complications were arterial hypertension (16.7%), MAS (10.0%), osteoporosis (8.3%), and diabetes mellitus (6.7%). The mean (SD) Pouchot score at initial diagnosis was 5.1 (2.0) on a scale of 0 to 12, indicating moderate disease activity (Table 1). Males and females had generally similar baseline characteristics, including age, but men had significantly higher Pouchot scores (with a mean [SD] of 3.8 [2.2] vs. 2.5 [2.2] for women; *p* < 0.001), indicating higher disease activity at the initial presentation.

### 3.1. Symptoms and Laboratory Findings: Initial Diagnosis vs. Current Status

**Figure 1:**
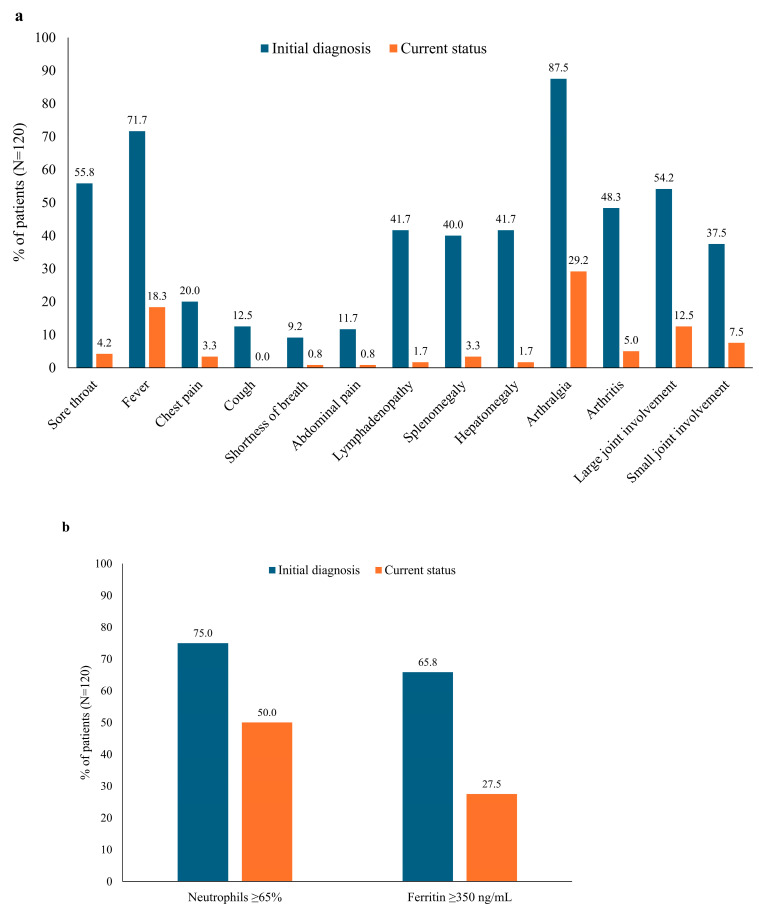
(**a**) Symptoms and (**b**) laboratory values at the initial diagnosis and most recent visit.

Arthralgia was the most frequent symptom at initial diagnosis (87.5%), and a substantial proportion of patients (29.2%) continued to experience arthralgia at the most recent follow-up (Figure 1a). Large-joint involvement was more common than small-joint involvement at both the initial diagnosis and follow-up. Fever was observed for 71.7% of patients at the initial diagnosis and 18.3% at the follow-up. Other common symptoms at the initial diagnosis, such as a sore throat, lymphadenopathy, splenomegaly, and hepatomegaly, had resolved in most patients at the most recent follow-up (Figure 1a). All disease symptoms showed significant reductions in prevalence between the initial diagnosis and the most recent follow-up (*p* < 0.001), except for arthralgia (*p* = 0.129) and fever (*p* = 0.178) (Table 2).

High neutrophil (accounting for ≥65% of white blood cells) and ferritin (≥350 ng/mL) levels were common in patients at the initial diagnosis (75.0% and 65.8%, respectively) (Figure 1b). Although both levels were reduced at the most recent follow-up, a substantial proportion of patients continued to experience high neutrophil or ferritin levels (50.0% and 27.5%, respectively), and the reduction in the proportion of patients with ferritin levels ≥350 ng/mL was not significant (*p* = 0.416) (Table 2). Significantly more females than males had high ferritin levels at the most recent follow-up (87% vs. 54%; *p* < 0.001).

### 3.2. Drug Therapies and Satisfaction with Treatment

**Figure 2:**
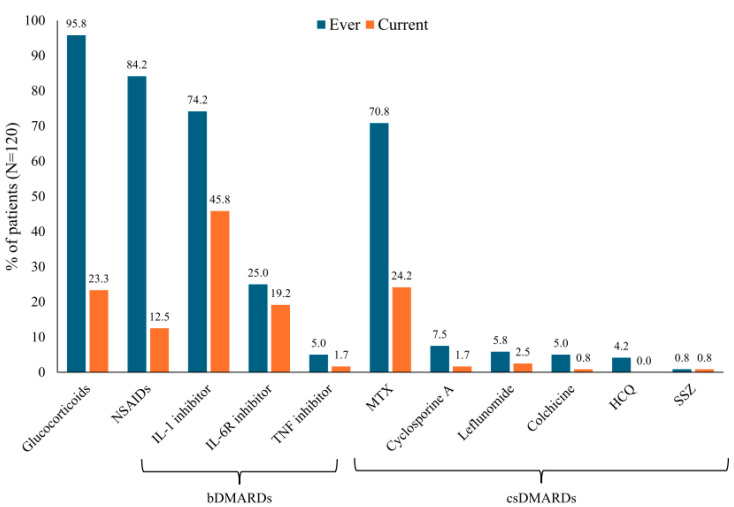
AOSD treatments received by patients at any time and at the current visit. bDMARD, biologic disease-modifying antirheumatic drug; csDMARD, conventional synthetic disease-modifying antirheumatic drug; HCQ, hydroxychloroquine; IL, interleukin; IL-6R, interleukin-6 receptor; MTX, methotrexate; NSAID, non-steroidal anti-inflammatory drug; SSZ, sulfasalazine, TNF, tumor necrosis factor.

Glucocorticoids and non-steroidal anti-inflammatory drugs (NSAIDs) had been used by almost all the patients at some point during the course of their disease (98.3% and 84.2%, respectively), but at the most recent follow-up, these drugs were used by only 23.3% and 12.5%, respectively (Figure 2). The two patients who had never received treatment with glucocorticoids were both managed using IL-1 inhibitors. IL-1 inhibitors were the most frequently used biologic disease-modifying antirheumatic drug (bDMARD) (with 74.2% having used it at any point and 45.8% using it at the follow-up), and MTX was the most frequently used csDMARD (with 70.8% having used it at any point and 24.2% using it at the follow-up). Over half of the patients currently on MTX (16/29 [55.2%]) received this therapy in combination with either an IL-1 or IL-6R inhibitor. IL-6R inhibitors were also used for some patients (with 25.0% having used it at any point and 19.2% using it at the follow-up). TNF inhibitors and csDMARDs other than MTX were less commonly used (current therapy for <5% of patients) (Figure 2).

Patient satisfaction with therapy at the last follow-up had a mean (SD) score of 6.63 (3.20) on a 10-point VAS scale ranging from 0 = worst to 10 = best (*n* = 99; no data were available for 21 patients). Twenty-three patients (19.2%) rated their satisfaction as 10, the highest level.

### 3.3. Disease Activity: Initial Diagnosis vs. Current Status

**Figure 3:**
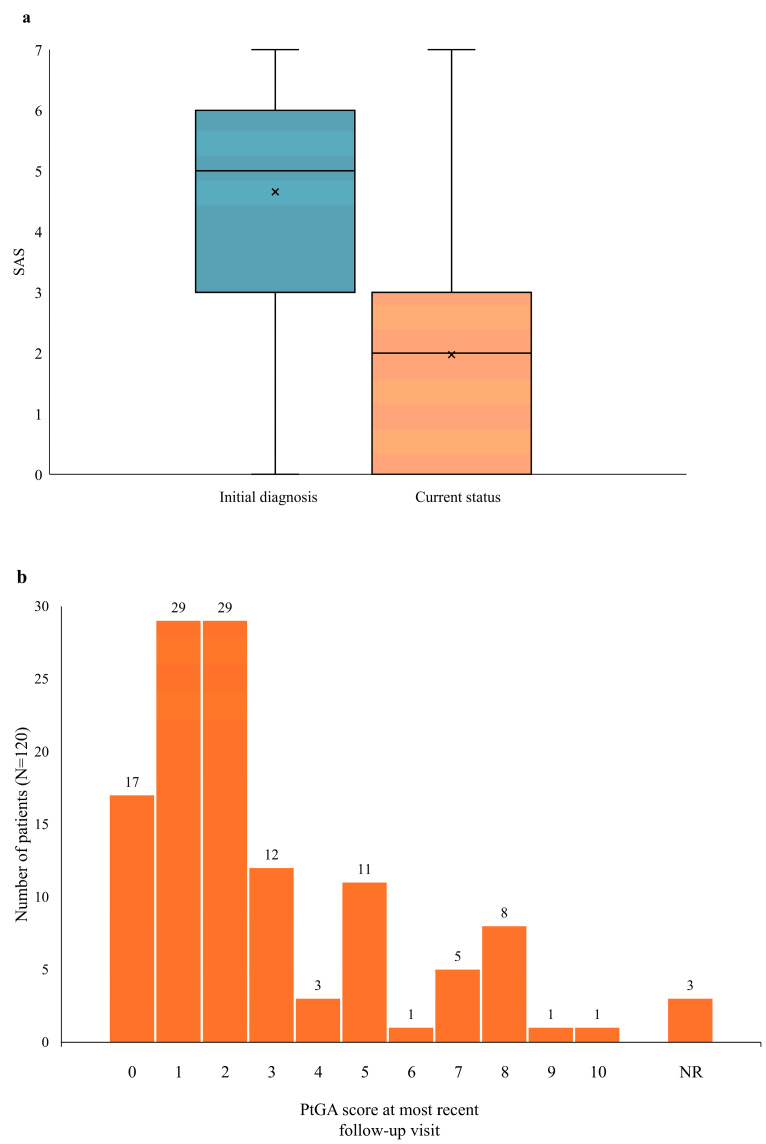
Disease activity scores. (**a**) Box-and-whisker plot of SAS at initial diagnosis and most recent follow-up. Boxes represent Q1 to Q3 values, the horizontal line indicates the median value, and the X indicates the mean value. Vertical lines indicate minimum and maximum data values. Data were missing for 1 patient at initial diagnosis and 5 patients at the current visit. (**b**) Histogram of PtGA scores at most recent follow-up visit (data were missing for 3 patients). SAS is scored on a scale of 0 to 7, and PtGA is scored on a VAS of 0–10. For both scales, lower scores indicate lower disease activity. NR, not reported; PtGA, patient assessment of global disease activity; SAS, Still’s Activity Score.

At the initial diagnosis, the mean (SD) SAS was 4.7 (1.9) on a scale of 0 to 7 (Figure 3a), consistent with the moderate disease activity indicated by the Pouchot score (5.1 [2.0]; Table 1). The SAS value fell to 2.0 (2.0) at the most recent follow-up (Figure 3a). More than one-quarter of the patients (34; 28.3%) were in remission (SAS = 0) at their most recent follow-up. Although the initial SAS values were similar between females and males, at the most recent follow-up, males had significantly higher mean SAS values (with a mean [SD] of 2.6 [2.3] vs. 1.5 [1.5] for women), indicating higher residual disease activity in males. Data were not available for the Pouchot score at the most recent visit, as this score is based in part on workups performed to exclude other conditions, which are not routinely performed. Although this study was not designed to evaluate the effect of treatment on disease activity, given the multiple confounding factors that could affect these assessments, such as age, baseline disease activity, and comorbidities, a preliminary analysis of SAS scores based on current therapy suggested there were similar SAS levels across drug classes (with a mean SAS of 1.50 for patients currently on MTX only, 1.85 for patients currently treated with IL-1 inhibitors, and 1.91 for patients currently treated with IL-6R inhibitors).

PtGA assessments were collected at the most recent follow-up but not at the initial diagnosis. These values indicated there was low disease activity for most patients at the current time (with a mean [SD] of 2.7 [2.5] on a 10-point VAS), with most patients having scores ≤ 2 (Figure 3b). Seventeen patients (14.2%) reported a PtGA score of 0. However, 10 patients (8.3%) reported PtGA scores ≥ 8, indicating that some patients continued to experience high disease activity. In general, there was good concordance between the PtGA scores and SAS values. For instance, all of the 17 patients with a PtGA score of 0 had an SAS of 2 or lower.

## 4. Discussion

This study of 120 patients with AOSD examined at German rheumatology centers documents the heavy comorbidity and symptom burden of this disease, particularly at the time of initial diagnosis. As has been observed in other cohorts [13,35,36,37], fever and arthralgia were the most common symptoms observed at the initial diagnosis, affecting 71.7% and 87.5% of patients, respectively. Although there were strong improvements in symptoms from the initial diagnosis to the visit during which data were collected, 18.9% of the patients still had a fever, and 29.2% had arthralgia. The high numbers of patients with elevated neutrophil (50.0%) and ferritin (27.5%) levels also suggested ongoing disease activity in some patients.

Consistent with the changes observed in symptoms, the SAS showed marked improvements in disease activity from initial diagnosis to current status. Based on the SAS, 28.3% of the patients were in remission when the data were reported. The PtGA values also supported low mean disease activity at the most recent visit for most patients. The PtGA scores at the most recent visit were generally concordant with the SAS scores.

Our findings indicate a high usage of MTX (70.8%) at the time of the initial diagnosis, a practice that is in line with the German Society of Rheumatology guidelines [32] but not considered the preferred treatment path according to the current EULAR/PReS guidelines [1] based on a systematic review of bDMARDs in AOSD [38]. IL-1 inhibitors were also used frequently at the time the initial diagnosis was made (74.2%) and were more likely to be reported as a treatment at the most current visit (45.8% vs. 24.2% for MTX). It should be emphasized that our study time period preceded the dissemination of the most recent guidelines on treatment management by these organizations, so it is likely that some of the treatment patterns we observed have changed in the intervening years. Part of our dataset also preceded the European Medicines Agency’s approval of using IL-1 inhibitors to treat AOSD (canakinumab in 2016 [39], anakinra in 2018 [40]), which may explain why only 90 out of 120 patients had ever undergone IL-1 inhibition. However, studies using data covering more recent years have also observed a high use of csDMARDs among AOSD patients. A retrospective study of 168 patients diagnosed with AOSD between 2007 and 2023 found that after the publication of the German AOSD guidelines, the time until a diagnosis was made was shorter, and treatment side effects were lower, but therapeutic approaches did not change substantially over time [16]. Similarly, a recent German study including data from 2007 through 2022 found that only about half of the included AOSD patients (44/86 [51.2%]) had received initial treatment with a bDMARD therapy [41]. These findings are consistent with a survey of 11 European AOSD experts (from Italy, the UK, France, and Germany) conducted in 2022, which found that most clinicians initiated treatment with glucocorticoids and used csDMARDs as a second-line treatment and bDMARDs as a third-line treatment [42].

Together, these findings on treatment patterns suggest that rheumatologists and other clinicians may require additional education on the changing therapeutic paradigms for AOSD, particularly with respect to IL-1 and IL-6R inhibitors as effective tools in the management of this disorder. It is hoped that new treatment strategies, including T2T approaches, could improve long-term outcomes [1,43]. A T2T approach to therapy has been proposed in the recent EULAR guidelines for AOSD [1] and is already considered the standard of care for sJIA [44,45,46] on the basis of studies indicating superior responses for sJIA patients treated with T2T strategies, particularly for those receiving biologic therapies, although head-to-head trials are lacking [47,48]. Although evidence for the use of T2T strategies in AOSD treatment has been lagged somewhat due in part to a lack of a consensus on outcome measures for response, given that sJIA and AOSD are now considered part of the same disease continuum, the application of T2T approaches to adults seems to be a reasonable extension of its use for pediatric patients. Furthermore, a recent study found that initial bDMARD therapy is associated with a more than 7-fold increase in the probability of achieving sustained, event-free remission compared with AOSD patients who do not receive initial bDMARD therapy [41]. These data suggest that early initiation of aggressive treatment along with a T2T approach may result in more favorable long-term outcomes.

The differences in the adoption of T2T in sJIA and AOSD are mirrored in other facets of disease management for these two disorders, including assessments of symptoms and treatment response. A recent literature review of 195 articles found wide heterogeneity in data reporting across the spectrum of sJIA and AOSD, and even within each disease type (sJIA or AOSD) [29]. Diagnostic criteria and disease activity measurements varied based on age and study, and reporting of symptoms and laboratory values lacked uniformity. For instance, arthralgia was evaluated in 12.0% of sJIA studies and 68.8% of AOSD studies. These variations are understandable given the relatively recent awareness that these two disorders were part of the same spectrum, but they still serve as a hindrance to the acquisition of a more complete picture of disease manifestations and response over time and to efforts to compare outcomes across different studies.

Along with standardized forms of assessment, the identification of conventional and novel biomarkers and their incorporation into diagnostic and therapeutic models may also help advance patient care [2,13]. Early studies suggest that the inclusion of biomarkers in outcome prediction models may improve evaluations of disease activity [49,50]. Although several biomarkers appear to be shared across sJIA and AOSD, particularly ferritin, S100 proteins, and IL-18 [2], additional studies are needed to assess the robustness of these indicators and the potential benefits of including them in clinical trials to assess responses to new therapies as well as in everyday clinical practice.

Educational efforts involving the prompt diagnosis of AOSD may also be of value. In Germany and elsewhere, the time from symptom onset to AOSD diagnosis has been reported to be longer than 1 year [13,16]. Delayed diagnosis or treatment may result in a missed therapeutic “window of opportunity”, potentially resulting in long-term damage [51,52]. In one study, AOSD patients with a delayed diagnosis (>6 months) were more likely to have a chronic disease course [36].

The limitations of this study include the low patient numbers, a fact related to the limited participation by German rheumatology centers, perhaps due to time constraints or inadequate reimbursement. The conclusions from this study were also limited by the retrospective study design and the use of only one visit for data collection. Patients with a monocyclic disease course may have been underrepresented in our study, as those with fully resolved disease may not have needed additional rheumatology visits. Our database did not include levels of inflammatory markers, such as CRP or ESR, or additional outcome measures, such as assessments of work impairment or different measures of patient satisfaction with treatment; these would be valuable additions for future studies. Because of the retrospective nature of this study, we were not able to perform further analyses of symptoms and outcomes associated with MAS and other significant complications of AOSD, including central nervous system involvement. Future surveys should endeavor to include additional information on serious disease manifestations and associated characteristics.

## 5. Conclusions

This retrospective study suggests that AOSD patients in Germany examined during routine clinical care show fewer symptoms and lower disease activity than they do at the initial diagnosis, but the symptomatic burden of disease remains high for some patients, and more advanced treatment options may be under-utilized. These findings may provide key “baseline” data for comparisons with future analyses of patient characteristics, disease activity, and outcomes following new treatment recommendations. In addition, future studies of outcomes and treatment responses for patients with serious complications of AOSD, particularly MAS; additional assessments of disease burden, including quality of life and other patient-reported outcomes; and longitudinal assessments of the course of Still’s syndrome are essential to provide a more complete picture of this complex disorder and its impact on patients.

## Figures and Tables

**Table 1:** AOSD patient characteristics. Data are presented as *n* (%) unless otherwise specified.

Characteristic	Value
N	120
Age at initial diagnosis, years ^a^	
Mean (SD)	41.4 (16.8)
Median (Q1, Q3)	40 (27, 55)
Age at most recent visit, years ^a^	
Mean (SD)	50.7 (16.2)
Median (Q1, Q3)	51 (36, 62)
Sex (as categorized by physician)	
Female	67 (55.8%)
Male	53 (44.2%)
Reason for visit	
Regular follow-up following earlier AOSD diagnosis	86 (71.7%)
Disease recurrence	34 (28.3%)
Disease course	
Monocyclic	25 (20.8%)
Polycyclic	66 (55.0%)
Chronic	29 (24.1%)
Current complications and comorbidities	
Hypertension	20 (16.7%)
MAS	12 (10.0%)
Osteoporosis	10 (8.3%)
Diabetes mellitus	8 (6.7%)
Time since diagnosis for most recent visit, years ^a^	
Mean (SD)	9.4 (6.9)
Median (Q1, Q3)	9 (4, 11)
Pouchot score at initial diagnosis ^a^	
Mean (SD)	5.1 (2.0)
Median (IQR)	5 (4, 6.5)

^a^ Data were missing for 1 patient. AOSD, adult-onset Still’s disease; MAS, macrophage activation syndrome; Q, quartile; SD, standard deviation.

**Table 2:** Statistical significance of changes in symptoms and disease activity between initial presentation and the most recent follow-up visit.

Outcome	*p* Value *
Signs and symptoms	
Abdominal pain	<0.001
Arthralgia	0.129
Cough	<0.001
Fever	0.178
Hepatomegaly	<0.001
Large-joint involvement	<0.001
Lymphadenopathy	<0.001
Shortness of breath	<0.001
Small-joint involvement	<0.001
Sore throat	<0.001
Splenomegaly	<0.001
Swollen joints	<0.001
Thoracic pain	<0.001
Measures of disease activity	
Ferritin > 350 ng/mL	0.416
Neutrophils > 65%	0.002
Still’s Activity Score	<0.001

* Statistical comparisons were conducted using McNemar’s Chi-squared test with continuity correction for all values, except Still’s Activity Score, for which the paired *t*-test was utilized.

## Data Availability

The original contributions presented in this study are included in the article. Further inquiries can be directed to the corresponding author.
